# Risk factors of immune checkpoint inhibitor-related interstitial lung disease in patients with lung cancer: a single-institution retrospective study

**DOI:** 10.1038/s41598-020-70743-2

**Published:** 2020-08-13

**Authors:** Naoto Okada, Rie Matsuoka, Takumi Sakurada, Mitsuhiro Goda, Masayuki Chuma, Kenta Yagi, Yoshito Zamami, Yasuhiko Nishioka, Keisuke Ishizawa

**Affiliations:** 1 Department of Pharmacy, Tokushima University Hospital, 2-50-1 Kuramoto, Tokushima, 770-8503 Japan; 2 Clinical Research Center for Developmental Therapeutics, Tokushima University Hospital, 2-50-1 Kuramoto, Tokushima, 770-8503 Japan; 3 Department of Clinical Pharmacology and Therapeutics, Tokushima University Graduate School of Biomedical Sciences, 3-8-15 Kuramoto, Tokushima, 770-8503 Japan; 4 Department of Respiratory Medicine and Rheumatology, Tokushima University Graduate School of Biomedical Sciences, 3-8-15 Kuramoto, Tokushima, 770-8503 Japan

**Keywords:** Oncology, Risk factors

## Abstract

Immune checkpoint inhibitors (ICIs) elicit antitumour effects by activating the host immunity and cause immune-related adverse events (irAEs). ICI-related interstitial lung disease (ICI-ILD) is a fatal irAE that is difficult to treat; moreover, its incidence is relatively higher in patients with lung cancer. Therefore, early ICI-ILD detection and intervention are important for patient safety. However, a risk assessment method for ICI-ILD has not been established and the prediction of ICI-ILD occurrence is difficult. The aim of our study was to identify the risk factors associated with ICI-ILD. To this end, we retrospectively analysed 102 patients with lung cancer who first received ICI and completed the treatment between April 2016 and December 2019 at Tokushima University Hospital. Nineteen patients had all grades of ICI-ILD and 10 had grade ≥ 3 ICI-ILD. The 30-day mortality rate of patients with grade ≥ 3 ICI-ILD was the highest among all patients (P < 0.01). The multivariate logistic analysis indicated that the performance status ≥ 2 alone and both performance status ≥ 2 and ≥ 50 pack-year were independent risk factors of ICI-ILD of grade ≥ 3 and all grades, respectively. Overall, our study provides insights to predict ICI-ILD occurrence.

## Introduction

Immune checkpoint inhibitors (ICIs) are antibodies that inhibit programmed death-1 (PD-1), PD ligand-1 (PD-L1), and cytotoxic T-lymphocyte-associated antigen-4 (CTLA-4), which are called immune checkpoint molecules. These molecules negatively regulate the host immunity; thus, the inhibition of these molecules activates the host immunity and exerts cross-organ antitumour effects^1^. In the treatment of lung cancer, some clinical trials have revealed that the administration of ICI alone and in combination with cytotoxic anticancer agents resulted in better clinical outcomes than previous standard treatments; thus, the use of ICI significantly improved lung cancer treatment^2–4^. However, the activation of the host immunity by ICI leads to characteristic adverse events, known as immune-related adverse events (irAEs), which exhibit profiles different from those caused by cytotoxic anticancer agents^5,6^. Therefore, the management of irAEs is essential for an effective ICI treatment.

These irAEs affect various organs, such as the skin, endocrine glands, gastrointestinal tract, and liver, but only a few events are fatal because the majority of them can be controlled by adequate treatment^5,7^. Specifically, ICI-related interstitial lung disease (ICI-ILD) has a low incidence (1–5%) and a high severity or mortality rate, according to clinical trials (50–60%)^8,9^. Patients with lung cancer are known to have relatively higher ICI-ILD rates than those with other cancers^10^. A prospective study of patients with lung cancer reported that the incidence of ICI-ILD was 14.5%, which is higher than that in clinical trials^11^. ICI-ILD has also been reported to affect the prognosis of patients with lung cancer^11,12^. Therefore, ICI-ILD onset is a limiting factor to continue or not continue ICI treatment and to achieve treatment benefits in patients with lung cancer. However, only a few studies have reported that the development of ICI-ILD was prevented^13^.

Proper clinical management of ICI-ILD requires the identification of patients who are at a high risk of developing ICI-ILD and the prevention of disease onset. Previous studies have shown that the expression of the anti-PD-1 antibody was higher than that of the anti-PD-L1 or anti-CTLA4 antibody in patients with ICI-ILD^14,15^. Furthermore, the incidence of ICI-ILD has been reported to be higher with the combination therapy of the anti-PD-1 and anti-CTLA4 antibodies than with monotherapy. Moreover, some studies have reported that pre-existing ILD in patients with lung cancer, whether induced by cytotoxic anticancer agents or ICI, is a risk factor for developing ILD^16–20^. In contrast, under pre-existing mild ILD, there was no increase in ICI-ILD frequency after treatment with nivolumab, an anti-PD-1 antibody^21^. Therefore, in the real-world setting, ICI treatment is limited to patients with no pre-existing or mild ILD to avoid the development of ICI-ILD. However, ICI-ILD also occurred in a group of patients with a low risk of ICI-ILD^21^, suggesting the existence of other unknown risk factors of ICI-ILD. Therefore, in our retrospective study, we aimed to identify risk factors associated with ICI-ILD.

## Results

### Characteristics of patients

**Figure 1:**
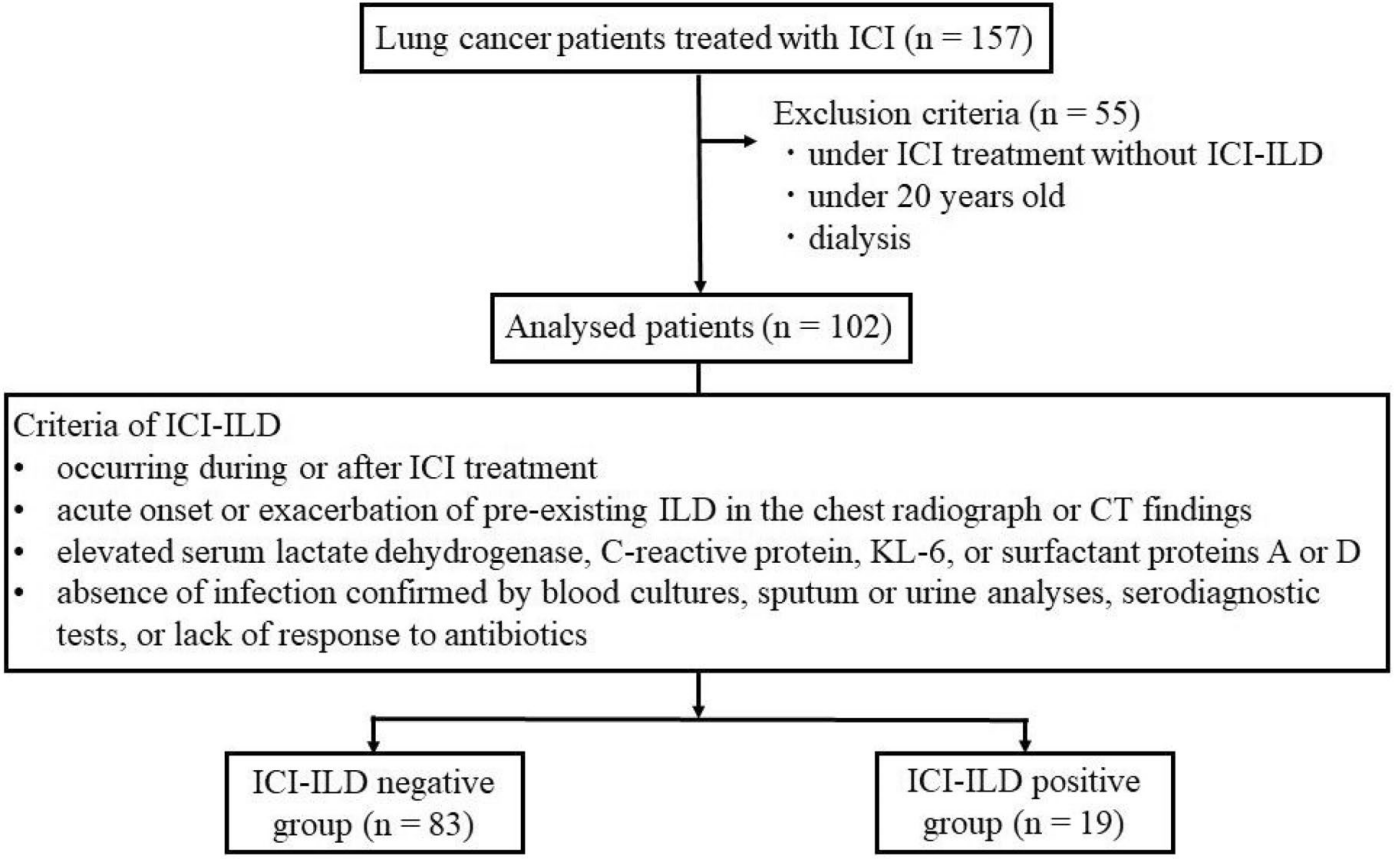
Study diagram. ICI: immune checkpoint inhibitor, ICI-ILD: immune checkpoint inhibitor-related interstitial lung disease, CT: computed tomography.

The study diagram is shown in Fig. 1. Of the 102 patients, 19 (18.6%) were diagnosed with ICI-ILD (ICI-ILD-positive group), and the rest were included in the ICI-ILD-negative group. None of the parameters—including sex, age, creatinine clearance, aspartate aminotransferase level, alanine aminotransferase level, albumin level, PD-L1 expression, pathology, clinical stage, driver mutation frequency, rate of patients who received antibodies against PD-1 or PD-L1, treatment line, previous treatment with epidermal growth factor receptor tyrosine kinase inhibitor (EGFR-TKI) or thoracic radiotherapy, and pre-existing chronic obstructive pulmonary disease (COPD) or ILD—differed between the ICI-ILD-positive and -negative groups (Table 1). Additionally, the patients did not receive ipilimumab, an anti-CTLA-4 antibody. Eleven patients had pre-existing ILD in both groups. Five patients had radiation-induced pneumonia, 4 had ILD with reticular shadow, 1 had drug-induced ILD, and 1 had usual interstitial pneumonia (UIP). All patients with pre-existing ILD were diagnosed with mild ILD because multiple computed tomography (CT) scans before ICI treatment showed no exacerbation of ILD and the clinical symptoms were mild. In contrast, the proportion of patients with Eastern Cooperative Oncology Group performance status (ECOG PS) of ≥ 2 in the ICI-ILD-positive group was higher than that in the negative group. Although there was no difference in smoking history between the two groups, the proportion of patients who consumed ≥ 50 pack-year was higher in the ICI-ILD-positive group than in the ICI-ILD-negative group.

**Table 1:** Characteristics of patients.

	ICI-ILD negative (n = 83)	ICI-ILD positive (n = 19)	P value
**Anti-PD-1 antibody**			
Nivolumab (%)	25 (30.1)	4 (21.1)	0.58
Pembrolizumab (%)	41 (49.4)	14 (73.7)	0.79
**Anti-PD-L1 antibody**			
Atezolizumab (%)	11 (13.3)	0 (0)	0.21
Durvalumab (%)	6 (7.2)	1 (5.3)	1.00
Sex (male) (%)	60 (72.3)	17 (89.5)	0.15
Age, median (range) (%)	69 (40–84)	70 (59–82)	0.13
Body weight (kg), median (range)	54.5 (30–88)	58 (39–75)	0.10
Creatinine clearance (mL/min), median (range)	64.3 (20.9–136.2)	67.7 (37.5–150.3)	0.36
Aspartate aminotransferase (IU/L), median (range)	22 (8–220)	22 (11–49)	0.59
Alanine aminotransferase (IU/L), median (range)	14 (3–120)	13 (6–49)	0.80
Albumin (g/dL), median (range)	3.6 (1.6–4.6)	3.8 (2.3–4.5)	0.38
**ECOG PS (%)**			0.02
< 2	74 (89.2)	13 (68.4)	
≥ 2	9 (10.8)	6 (31.6)	
**Pathology (%)**			0.43
Adenocarcinoma	54 (65.1)	12 (63.2)	
Squamous	13 (15.7)	5 (26.3)	
Other	16 (19.3)	2 (10.5)	
**Stage (%)**			0.55
III	17 (20.5)	5 (26.3)	
IV	66 (79.5)	14 (73.7)	
**Driver mutation (%)**			
EGFR	4 (4.8)	0 (0)	1.00
ALK	0 (0)	0 (0)	
**PD-L1 status (%)**			0.59
< 1%	36 (43.4)	7 (26.8)	
> 50%	29 (34.9)	9 (47.4)	
Unknown	18 (21.7)	3 (15.8)	
**Treatment line (%)**			0.58
First	21 (25.3)	6 (31.6)	
Second or higher	62 (74.7)	13 (68.4)	
**History of smoking (%)**	66 (79.5)	18 (94.7)	0.18
≥ 30 pack-year (%)	51 (61.4)	15 (78.9)	0.19
≥ 50 pack-year (%)	29 (34.9)	12 (63.2)	0.02
Previous EGFR-TKI (%)	4 (4.8)	0 (0)	1.00
Previous thoracic radiotherapy (%)	23 (27.7)	7 (36.8)	0.43
Pre-existence of COPD (%)	21 (25.3)	9 (47.4)	0.06
Pre-existence of ILD (%)	9 (10.8)	2 (10.5)	1.00

ICI-ILD: immune checkpoint inhibitor-related interstitial lung disease, PD-1: programmed death-1, PD-L1: programmed death-1 ligand, ECOG PS: Eastern Cooperative Oncology Group performance status, EGFR: epidermal growth factor receptor, ALK: anaplastic lymphoma kinase, TKI: tyrosine kinase inhibitor, COPD: chronic obstructive pulmonary disease.

### Outcome assessment

The profile of patients with ICI-ILD is shown in Table 2. The number of patients who developed ICI-ILD during 1, 2, 3, 4, and ≥ 5 courses of ICI treatment was 5, 3, 0, 1, and 10, respectively. In contrast, 4, 5, 6, 0, and 4 patients developed ICI-ILD of grades 1, 2, 3, 4, and 5, respectively, according to the Common Terminology Criteria for Adverse Events (CTCAE) ver.5.0. Chest CT of 11 patients with ICI-ILD showed a ground-glass attenuation pattern. Supplementary Fig. 1 shows the CT images of representative patients with grade 1, 3, and 5 ICI-ILD. Patients received different treatments according to the ICI-ILD grade. Patients with grade 1 ICI-ILD were not treated. However, patients with grade 2 ICI-ILD received oral steroids. Oral or intravenous steroid therapies were administered to patients with grade 2 and > 3 ICI-ILD, respectively. Infliximab or cyclophosphamide was administered to patients with severe ICI-ILD. In 15 patients who received immunosuppressive therapy for ICI-ILD, four patients died within 30 days of treatment initiation. After the immunosuppressive therapy, while one patient showed no changes in symptoms or in CT imaging, the remaining patients showed an improvement in clinical symptoms and in CT imaging; there was no recurrence of ILD during the analysis period in all patients. The 30-day mortality of patients with grade ≥ 3 ICI-ILD was significantly higher than that of patients without ICI-ILD (Supplementary Fig. 2a). In contrast, there were no differences in the overall survival rate among the ICI-ILD-negative, grade 1 or 2 ICI-ILD, and grade ≥ 3 ICI-ILD groups (Supplementary Fig. 2b). The ICI-ILD-positive group had a significantly higher incidence of other irAEs, such as skin rash, than the negative group (Table 3).

**Table 2:** Profile of patients with ICI-ILD.

Characteristics	ICI-ILD positive (n = 19)
**Number of ICI cycles**	
1/2/3/4/ ≥ 5	5/3/0/1/10
**CTCAE grade**	
1/2/3/4/5	4/5/6/0/4
**CT findings of ICI-ILD (%)**	
Ground-glass opacity	11 (57.9)
Consolidation	7 (36.8)
Reticular shadow	3 (15.8)
Traction bronchiectasis	2 (10.5)
**Treatment of ICI-ILD (%)**	
None	4 (21.1)
Oral steroids	6 (31.6)
Pulse steroids	5 (26.3)
Intravenous steroids	4 (21.1)
Infliximab	1 (5.3)
Cyclophosphamide	1 (5.3)

ICI: immune checkpoint inhibitor, ICI-ILD: immune checkpoint inhibitor-related interstitial lung disease, CT: computed tomography, CTCAE: Common Terminology Criteria for Adverse Events.

**Table 3:** Relationship between ICI-ILD diagnosis and other irAEs.

	ICI-ILD negative (n = 83)	ICI-ILD positive (n = 19)	P value
irAE negative (%)	55 (66.3)	6 (31.6)	< 0.01
**irAE positive (%)**	28 (33.7)	13 (68.4)	
Rash	11	5	
Hypothyroidism	9	1	
Destructive thyroiditis	0	1	
Adrenal insufficiency	2	1	
Liver dysfunction	2	2	
Renal dysfunction	1	1	
Diarrhoea	0	2	
Other	3	0	

ICI-ILD: immune checkpoint inhibitor-related interstitial lung disease, irAE: immune-related adverse event.

### Factors associated with ICI-ILD

**Figure 2:**
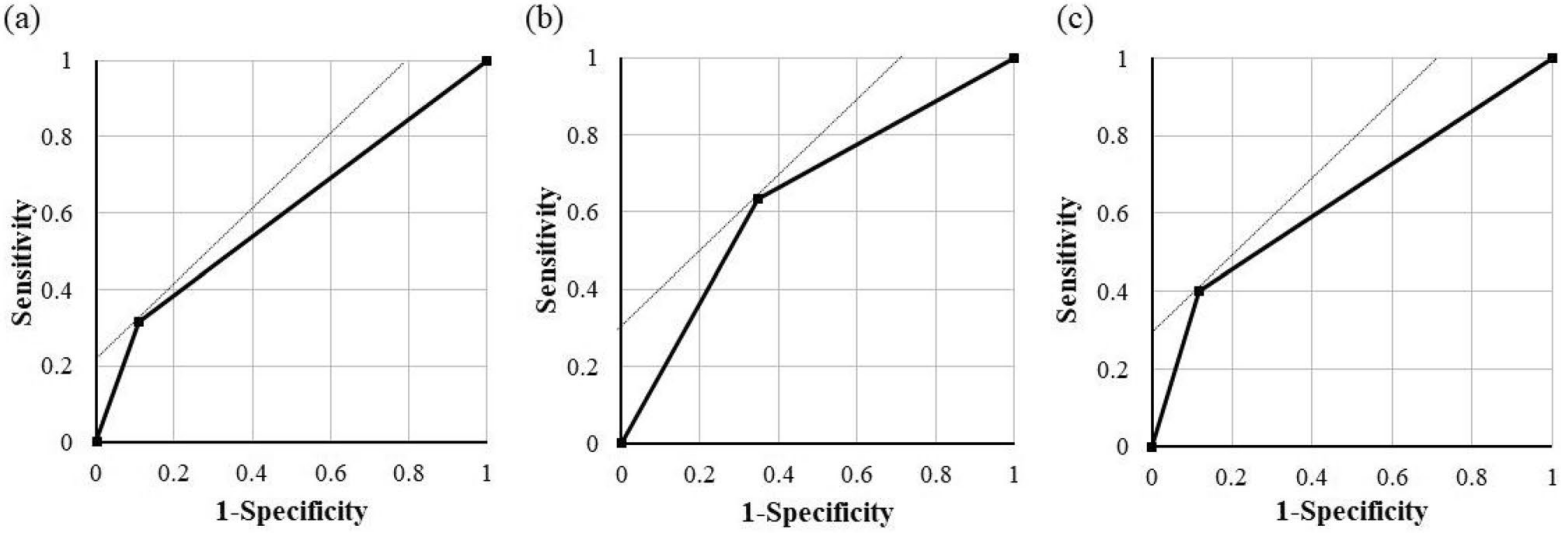
ROC curves for PS and pack-year to estimate prediction accuracy. The ROC curve of (**a**) ECOG PS ≥ 2 and (**b**) ≥ 50 pack-year including all ICI-ILD and (**c**) ECOG PS ≥ 2 including ICI-ILD grade ≥ 3. ROC: receiver operating characteristic, ICI-ILD: immune checkpoint inhibitor-related interstitial lung disease, ECOG PS: Eastern Cooperative Oncology Group performance status.

Table 4 shows the results of the univariate and multivariate logistic regression analyses that were performed to identify the risk factors of ICI-ILD. The univariate regression analysis including patients with ICI-ILD of all grades only revealed significant differences in the subgroups of ECOG PS ≥ 2 and ≥ 50 pack-year. The other factors were not different in the univariate analysis. Multivariate model 1 and 2 analyses confirmed that ECOG PS ≥ 2 and ≥ 50 pack-year were independent risk factors associated with ICI-ILD of all grades. The receiver operating characteristic (ROC) curve analysis of ECOG PS ≥ 2 including all ICI-ILD grades yielded a sensitivity of 31.5%, specificity of 89.2%, and an area under the ROC curve (AUC) of 0.61 (Fig. 2a). In contrast, the ROC curve analysis of ≥ 50 pack-year including all ICI-ILD grades indicated a sensitivity of 63.2%, specificity of 65.2%, and an AUC of 0.65 (Fig. 2b). Moreover, the univariate and multivariate model 1 and 2 analyses revealed that only ECOG PS ≥ 2 was the risk factor of grade ≥ 3 ICI-ILD. The ROC curve analysis of ECOG PS ≥ 2 for grade ≥ 3 ICI-ILD showed a sensitivity of 40.0%, specificity of 88.0%, and an AUC of 0.64 (Fig. 2c).

**Table 4:** Univariate and multivariate logistic regression analyses.

Variable	Univariate			Multivariate model 1			Multivariate model 2		
	OR	95% CI	P value	OR	95% CI	P value	OR	95% CI	P value
**All grade ICI-ILD**									
ECOG PS ≥ 2	3.79	1.16–12.47	0.028	3.43	1.01–11.71	0.048	3.44	1.01–11.80	0.048
≥ 50 pack-year	3.19	1.13–8.99	0.028	2.97	1.03–8.56	0.043	3.01	1.04–8.72	0.042
Pre-existing ILD	0.97	0.19–4.49	0.97	–	–	–	0.78	0.14–4.24	0.77
**Grade ≥ 3 ICI-ILD**									
ECOG PS ≥ 2	4.91	1.19–20.17	0.027	4.34	1.02–18.60	0.048	4.36	1.02–18.71	0.048
≥ 50 pack-year	3.98	0.96–16.42	0.056	3.59	0.84–15.27	0.084	3.65	0.85–15.57	0.084
Pre-existing ILD	0.91	0.10–7.96	0.93	–	–	–	0.72	0.075–6.88	0.77

ICI-ILD: immune checkpoint inhibitor-related interstitial lung disease, ECOG PS: Eastern Cooperative Oncology Group performance status, OR: odds ratio, CI: confidential interval.

## Discussion

Our study showed that ECOG PS ≥ 2 alone and both ECOG PS ≥ 2 and ≥ 50 pack-year were the risk factors of ICI-ILD of grade ≥ 3 and all grades, respectively.

The rate of ICI-ILD was 18.6%, which is higher than that reported in previous clinical studies^8,9^, but similar to that of real-world studies^19,20^. In a prospective study of Japanese patients, the incidence of ICI-ILD was 14.5%, which is consistent with our finding^11^. This higher rate of ICI-ILD in a real-world setting could be due to the fact that patients with poorer lung conditions were treated with ICI but excluded from clinical trials. Moreover, a previous study reported that Japanese patients exhibited a higher frequency of drug-induced ILD than other races^22^. The higher expression rates observed in the present study may be because we analyse only the Japanese population.

The mechanism that modulates ICI-ILD onset has not been fully elucidated. However, increased levels of inflammatory cytokines may be involved in the pathophysiology of irAE^23,24^. The inflammatory cytokine interleukin (IL)-6 induces the differentiation of naive CD4 T cells to Th17 cells, and it has been related to the incidence of irAEs^25^. Th17 cells are key mediators of several autoimmune diseases, some of which are irAEs, by producing IL-17^26,27^. A recent study on the beneficial effect of tocilizumab, an IL-6 antibody, in the treatment of irAE suggested that inflammatory cytokines, such as IL-6 and IL-17, are involved in ICI-induced irAEs^28^. Similarly, tumour necrosis factor-α (TNF-α) has been implicated in ICI-induced irAEs, and the use of anti-TNF-α antibodies for severe irAEs has been considered^29^. The levels of inflammatory cytokines, such as IL-6 and TNF-α, increased in patients with poor PS and cancer cachexia^30,31^. Thus, patients with a poor PS may be susceptible to ICI-induced irAEs due to high levels of intrinsic inflammatory cytokines, such as IL-6, IL-17, and TNF-α. Because a poor PS has been implicated in the development of grade ≥ 3 ICI-ILD, ICI treatment of patients with a poor PS should be avoided.

Although smoking history was not identified as a risk factor of ICI-ILD in this study, unlike drug-induced ILD^13^, ≥ 50 pack-year was associated with the development of ICI-ILD of all grades. This result suggested that when treating lung cancer with ICI, the presence or absence of smoking history and quantitative assessments of smoking should be considered. Additionally, heavy smoking can affect the lung before ICI treatment and lead to chronic respiratory diseases, such as atelectasis and COPD^32^. In this analysis, more patients in the ICI-ILD positive group tended to have a history of COPD (45% vs. 25%, P = 0.06). ICI treatment of patients with poor lung conditions can easily lead to ICI-ILD. Conversely, smoking history was considered a prognostic marker for ICI treatment^33^. DNA damage-induced by smoking may favour ICI treatment. Patients with lung cancer who smoked > 20 pack-year exhibited mutations in gene products associated with a favourable response to ICI therapy^33^. Taken together, smoking may be a favourable marker for ICI treatment or a risk factor of ICI-ILD, depending on its frequency. Patients who smoke heavily may benefit from the treatment, and they are at a higher risk for developing ICI-ILD.

In the present study, all patients with pre-existing ILD had mild ILD. The univariate and multivariate analyses did not identify pre-existing mild ILD as a risk factor of ICI-ILD. Previous studies have shown that symptomatic mild UIP is not a risk factor of ICI-ILD^21^. Although one patient with pre-existing ILD was diagnosed with mild UIP, the other patients were diagnosed with other types of mild ILD because their clinical symptoms were mild before ICI treatment, and their CT images before ICI showed no exacerbation of ILD. This indicated that inactive pre-existing mild ILDs, including UIP, may not be a risk factor of ICI-ILD. We should evaluate the severity of pre-existing ILD, not its presence or absence, when determining if ICI treatment is appropriate for a certain patient in order to obtain the beneficial outcomes of ICI treatment.

In this study, a higher proportion of anti-PD-1 antibody users developed ICI-ILD than anti-PD-L1 antibody users (5.5% vs. 21.4%, P = 0.18). This trend is consistent with those reported previously^14,15^. Because previous studies showed that the type of ICI was not a risk factor of ICI-ILD, the effect of ICI type on the incidence of ICI-ILD may be negligible. During the analysis, because the combination of the anti-PD-1 and anti-CTLA4 antibodies was not available in Japan, we could not analyse the effect of the ICI combination on ICI-ILD occurrence. The difference in the occurrence of ICI-ILD under treatments with anti-PD-L1 antibody and anti-PD-1 antibody might be influenced by the differential inhibition of interaction between PD-1 and PD ligand-2 (PD-L2)^15^. Further analysis of differences in the effects of different types of ICI on ICI-ILD is needed.

Interestingly, patients with ICI-ILD showed a higher rate of concomitant irAEs than patients without ICI-ILD (Table 3). In this study, ICI-ILD frequently developed during the first or second course of ICI treatment, similar to the onset timing of other irAEs^29^. This result indicates that we should pay attention to the overlapping development of ICI-ILD and other irAEs. In ICI-treated patients with lung cancer, irAEs were reported as a predictive marker related to increased overall survival^34^. However, the 30-day mortality of patients with grade ≥ 3 ICI-ILD was significantly higher than that of other patients (Supplementary Fig. 2). Thus, severe ICI-ILD may not be a predictive marker of overall survival prolongation in patients with lung cancer. Previous studies have reported the prognostic effect of ICI-ILD, but it is still controversial^11,12^. A recent prospective study reported that ICI-ILD had no effect on prognosis^11^. However, the effect of severe ICI-ILD alone has not been discussed. The results of our study suggest that severe ICI-ILD may worsen the prognosis. In contrast, there was no difference in the 30-day mortality rate between patients with ICI-ILD grade 1 or 2 and no ICI-ILD (Supplementary Fig. 2). Moreover, prolonged overall survival was observed in patients with irAEs, including grade 1–2 ICI-ILD, compared with patients without irAEs (hazard ratio: 0.46, 95% CI: 0.23–0.93). This finding indicated that appropriate therapeutic interventions for mild ICI-ILD are important for a favourable ICI treatment. In our cohort, 0.5–1 mg/kg oral steroid therapy was administered to patients with mild ICI-ILD resulting in a good response; recurrence of ILD was not observed in any patient during the observation period. Therefore, to prevent severe ICI-ILD, (1) patients who can be treated with ICI should be selected based on risk factors, (2) CT thresholds should be lowered in patients at a high risk of ICI-ILD, and (3) appropriate therapeutic intervention should be provided.

There were a few limitations to our study. First, the sample size was small, and therefore, the evaluated statistical power might be insufficient. Particularly, because the number of patients who developed severe ICI-ILD was small, leading to type 2 error, further analyses with more patients are needed. Second, owing to the small number of patients analysed, the prediction accuracy of the identified risk factors was still inadequate. However, when PS ≥ 2 and ≥ 50 pack-year were evaluated in combination, the sensitivity, specificity, and AUC of all ICI-ILD grades were increased to 73.7%, 59.0%, and 0.69, respectively; thus, we recommend the use of both factors in future analysis. Third, because we did not perform lung tissue diagnosis at the time of death, it is unclear whether the cause of 30-day mortality is an ineffective immunosuppressive therapy and an exacerbation of ILD or progression of malignant disease. Fourth, in this analysis, the diagnosis of ILD was made by imaging and examining clinical symptoms and blood markers, but ICI-ILD was not diagnosed by histological assessment. Therefore, we should not rule out the possibility that ICI-ILD was present even in patients without a diagnosis of ICI-ILD according to our criteria. Finally, because the follow-up period of this study was short, a detailed assessment of the overall survival of patients with ICI-ILD requires further validation.

In conclusion, our study demonstrated that PS ≥ 2 is a risk factor of grade ≥ 3 ICI-ILD and both PS ≥ 2 and ≥ 50 pack-year were independent risk factors of ICI-ILD of any grade. Although further analyses are required to confirm our findings, we showed the importance of preventing ICI-ILD onset, a fatal irAE hindering ICI treatment. Overall, our study provides insights to predict ICI-ILD occurrence.

## Methods

### Study design and population

We analysed 157 patients with lung cancer who first received nivolumab or pembrolizumab (anti-PD-1 antibody), atezolizumab or durvalumab (anti-PD-L1 antibody) between April 2016 and December 2019 at Tokushima University Hospital. In Japan, ipilimumab (anti-CTLA-4 antibody) and avelumab (anti-PD-L1 antibody) were not approved for lung cancer treatment during this study period. Patients (n = 55) were excluded from the study because their ICI treatment was continued beyond the observation period. No patient underwent renal replacement therapy or was less than 20 years old. Patients with lung cancer who were eligible for this study (n = 102) were divided into two groups, ICI-ILD-positive and -negative groups. The following clinicopathological factors were retrospectively obtained from their medical charts: sex, age, body weight, creatinine clearance, aspartate aminotransferase level, alanine aminotransferase level, albumin level, pathology, clinical cancer stage, ECOG PS, driver mutation frequency, PD-L1 expression, treatment line, previous use of EGFR-TKI, previous treatment with thoracic radiotherapy, pre-existing COPD or ILD, smoking history, and smoking status (in terms of pack-year). Pre-existing ILD was diagnosed using clinical symptoms and by chest CT imaging before ICI administration. IrAEs were defined according to a previous report^6^. Creatinine clearance was calculated according to previously described methods^35^. Pack-year was calculated as the number of cigarettes smoked per day × smoking year/20.

### Outcome assessment

ICI-ILD was defined according to a previous study; (1) occurring during or after ICI treatment, (2) acute onset or exacerbation of pre-existing ILD according to chest radiography or CT findings, (3) elevated serum lactate dehydrogenase, C-reactive protein, KL-6, or surfactant protein A or D level, and (4) absence of infection confirmed by blood cultures, sputum or urine analysis, serodiagnosis, or lack of response to antibiotics^18^. For patients with ICI-ILD, the grade and treatment of ICI-ILD and the chest CT pattern were assessed. The grades of ICI-ILD and other irAEs were assessed using CTCAE version 5.0. The 30-day mortality rate was defined as the rate of death by any cause within 30 days after ICI treatment. The efficacy of immunosuppressive therapy for ICI-ILD was assessed by clinical symptoms or radiographic test. Overall survival was defined based on the period between ICI treatment initiation and patient death or last follow-up.

### Statistical analysis

Differences between the two groups were analysed using parametric unpaired *t*-test or nonparametric Mann–Whitney U test, as applicable. Data distribution was evaluated using Shapiro–Wilk test and visualisation of histograms. The chi-square or Fisher’s exact test was to analyse the nominal scales. Univariate and multivariate logistic regression models were used to identify the risk factors associated with ICI-ILD. In the multivariate analysis, we used two models for a more accurate assessment of the risk factors. In model 1, factors possibly associated with ICI-ILD were identified using the univariate analysis (P < 0.05), and they were used in the multivariate analysis by forced entry method. In model 2, in addition to the factors identified using the univariate analysis (P < 0.05), pre-existing ILD, which was identified as a risk factor by previous studies, was also used in the multivariate analysis by the forced entry method. The ROC curve analysis was performed to calculate the AUC to confirm the diagnostic values of the selected variables. Survival curves were obtained using the Kaplan–Meier method and were compared using the log-rank test. For all statistical analyses, values of P < 0.05 (two-tailed) were considered significant. All analyses were performed using JMP version 14.3.0 (SAS Institute, Cary, NC, USA).

### Ethical approval statement

The study protocol was approved by the Tokushima University Hospital Ethics Committee and was conducted in accordance with the stipulations on the handling of patients’ personal information (Ethics Committee Registration Number: 3566). Owing to the retrospective nature of this study, informed consent was waived and our official website was used as an opt-out method, which was approved by the Tokushima University Hospital Ethics Committee.

## Supplementary information

Supplementary Information

## Data Availability

Correspondence and requests for materials should be addressed to N. O.
