# Supplementary material for: Risk factors of immune checkpoint inhibitor-related interstitial lung disease in patients with lung cancer: a single-institution retrospective study
Source: Sci Rep. 2020 Aug 13;10:13773. doi: 10.1038/s41598-020-70743-2 (PMC7426925; doi:10.1038/s41598-020-70743-2)

**Risk factors of immune checkpoint inhibitor-related interstitial lung disease in patients with lung cancer: a single-institution retrospective study**

**Authors: Naoto Okada, Rie Matsuokaa, Takumi Sakurada, Mitsuhiro Goda, Masayuki Chuma, Kenta Yagi, Yoshito Zamami, Yasuhiko Nishioka, Keisuke Ishizawa**

**Supplementary Figure 1. Representative CT images of patients with ICI-ILD**

Each CT image is an image of a separate slice of the same patient. Grade 1 patient’s CT showed focal lesions of ground-glass attenuation. Grade 3 patient’s CT showed consolidation with ground-glass attenuation in the left lung. Grade 5 patient’s CT showed widespread ground-glass attenuation with traction-bronchiectasis in bilateral lungs.

**Supplementary Figure 2. Endpoint assessment of ICI-ILD**

Thirty-day mortality rate (**a**) and Kaplan–Meier plot (**b**) classified by ICI-ILD severity. Black dots show censored cases lost to follow-up.

Abbreviations: ICI-ILD: immune checkpoint inhibitor-related interstitial lung disease, G: grade, N. S.: not significant

**Supplemental Figure 1.** Representative CT images of patients with ICI-ILD in this study


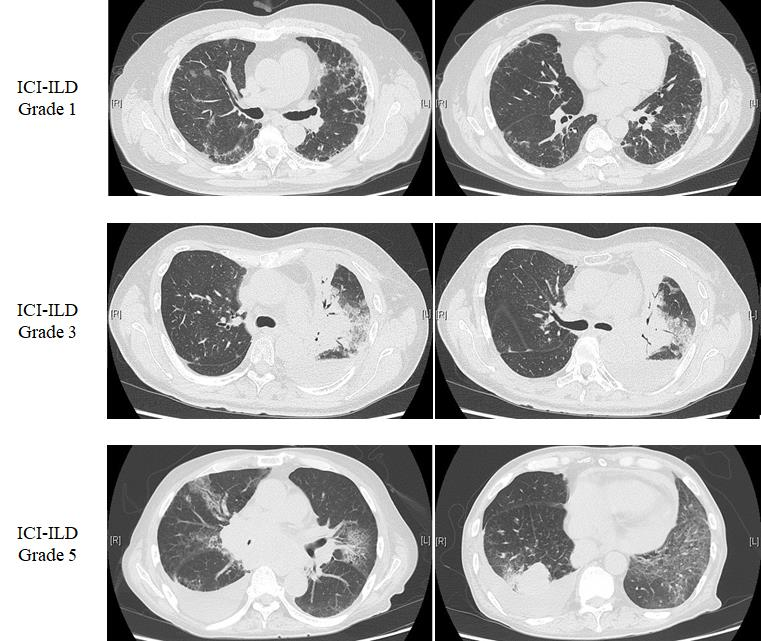


**Supplemental Figure 2.** Endpoint assessment of immune checkpoint inhibitor-related interstitial lung disease (ICI-ILD)


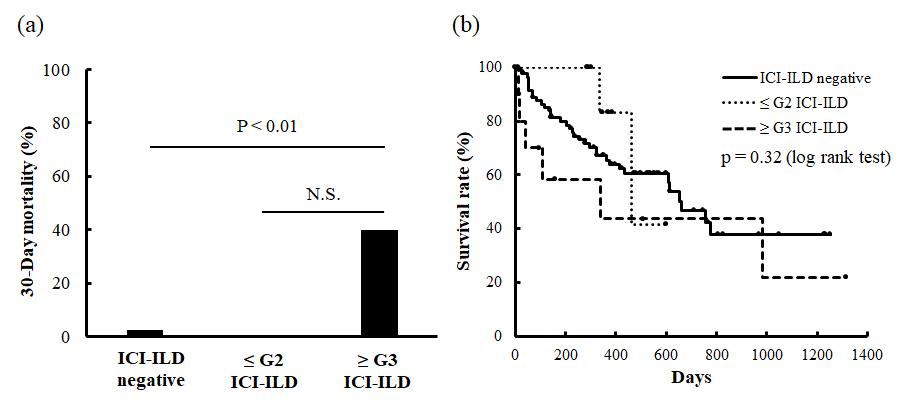

Supplement: Supplementary file 1 — Supplementary Information [file 41598_2020_70743_MOESM1_ESM.docx]
